# Computational and experimental studies of a cell-imprinted-based integrated microfluidic device for biomedical applications

**DOI:** 10.1038/s41598-021-91616-2

**Published:** 2021-06-09

**Authors:** Sepideh Yazdian Kashani, Mostafa Keshavarz Moraveji, Shahin Bonakdar

**Affiliations:** 1 Department of Chemical Engineering, Amirkabir University of Technology (Tehran Polytechnic), Tehran, 1591634311 Iran; 2 National Cell Bank Department, Pasteur Institute of Iran, P.O. Box 13169-43551, Tehran, Iran

**Keywords:** Biological techniques, Stem cells, Medical research, Engineering

## Abstract

It has been proved that cell-imprinted substrates molded from template cells can be used for the re-culture of that cell while preserving its normal behavior or to differentiate the cultured stem cells into the template cell. In this study, a microfluidic device was presented to modify the previous irregular cell-imprinted substrate and increase imprinting efficiency by regular and objective cell culture. First, a cell-imprinted substrate from template cells was prepared using a microfluidic chip in a regular pattern. Another microfluidic chip with the same pattern was then aligned on the cell-imprinted substrate to create a chondrocyte-imprinted-based integrated microfluidic device. Computational fluid dynamics (CFD) simulations were used to obtain suitable conditions for injecting cells into the microfluidic chip before performing experimental evaluations. In this simulation, the effect of input flow rate, number per unit volume, and size of injected cells in two different chip sizes were examined on exerted shear stress and cell trajectories. This numerical simulation was first validated with experiments with cell lines. Finally, chondrocyte was used as template cell to evaluate the chondrogenic differentiation of adipose-derived mesenchymal stem cells (ADSCs) in the chondrocyte-imprinted-based integrated microfluidic device. ADSCs were positioned precisely on the chondrocyte patterns, and without using any chemical growth factor, their fibroblast-like morphology was modified to the spherical morphology of chondrocytes after 14 days of culture. Both immunostaining and gene expression analysis showed improvement in chondrogenic differentiation compared to traditional imprinting methods. This study demonstrated the effectiveness of cell-imprinted-based integrated microfluidic devices for biomedical applications.

## Introduction

It has been proven that cells’ natural environment effectively controls cells’ function, and cells lose their normal behavior after isolating from their natural environment^1–3^. For example, chondrocytes’ spherical morphology will be lost after a mono-layer culture on a polystyrene plate, and they will gain fibroblast-like morphology^4^. Also, providing healthy chondrocytes from the patient or donor and in vitro culture for knee osteoarthritis treatment is challenging^5,6^. Many researchers have been attracted to adipose-derived mesenchymal stem cells (ADSCs), which can be differentiated for generating cartilage to overcome the limitations of using chondrocytes^5,7,8^.

In regenerative medicine based on stem cell manipulation, researchers try to imitate the cells’ natural environment by creating similar conditions for their growth and differentiation. The role of culture substrate in cellular behavior has been evaluated in several studies^9–12^. Also, researchers have confirmed the importance of surface topography for stem cell differentiation^13–18^.

The method of inducing differentiation in stem cells through cell shape engineering (imprinting) was first implemented by Mahmoudi et al.^2^. A cell-imprinted substrate was fabricated from the chondrocyte shape as a physical stimulus for inducing chondrogenic differentiation in stem cells. Cardiomyogenic, tenogenic, osteogenic, kertainogenic, and Schwann cell differentiation in stem cells were also obtained with the imprinting method^19–24^. Also, the effect of physical topography on the cancer cells’ response to the conventional anti-cancer drugs was investigated in^25^. Cancer bioimprinting and cell shape recognition can improve detection limits or eliminate the need for a thorough patient samples analysis^26^. So, the cell-imprinted substrate can manipulate cell phenotypes and regulate their function^27,28^. However, this process’s efficacy is poor because of the lack of control over the cells’ location.

The rapid development of microfluidic technology is a way of mimicking an in vivo-like microenvironment^29–32^. Microfluidic devices’ other benefits are biocompatibility, high surface-area-to-volume ratio, continuous and homogenous feeding of cells, automated cell culture media perfusion, and ease of handling. Microfluidic devices are used with the controlled microenvironment to investigate the effects of external factors on cell fate^5,33–38^. Therefore, many research efforts are now focused on using microfluidic devices for cell culture studies in developing medicines and biological research applications, such as drug toxicity or metabolism studies, also for stem cell research^6,29,39–42^. Stem cell culture and differentiation require careful control of several cell culture microenvironment signals regulating intracellular signaling and, eventually, cell phenotype. The development of such precise monitoring is difficult for traditional cell culture systems^29,43^. In addition, in comparison with conventional methods, microfluidic systems can simultaneously combine physical and biochemical factors to provide precise and repeatable stimulation for controlled stem cell differentiation, which is very important in regenerative medicine^29,43–51^.

In traditional imprinting methods, the template cells have entirely random placement, so the secondary cell’s probability of being placed exactly on the first cell template is low. Therefore, herein to increase the traditional imprinting methods efficiency, a microfluidic-based platform is introduced. The template cells are first cultured in a microfluidic chip on a cell culture plate. Their topography is transferred to a silicone replica by mold casting in a regular pattern. The regular cell-imprinted pattern is then used as a second culture substrate under the other microfluidic chip aligned to the regular cells pattern. The cell culture environment is both predictable and controllable since the entire process is performed inside the chips.

Furthermore, cells’ culture is dynamic, and the cell culture medium passes continuously over cells, mimicking the fluidic flow like in the body. Using a micrometer level cell-imprinted-based integrated microfluidic device reduces the number of cells needed in one experiment. It introduces the sufficiently accurate, reproducible, and low-cost substitute of traditional cell culture plates to control cells’ fate. This procedure can be used in cell therapy or drug analysis while preserving normal cell activity or stem cell differentiation into target cells.

Although these experimental methods are reliable, they are very time-consuming to characterize the fluid flow in a microfluidic cell chip. By means of computational fluid dynamics (CFD), certain parameters such as fluid inlet velocity and its effect on shear stress applied to cells or injection cell concentrations and its effect on filling microfluidic chip microchannels can also be evaluated to predict their effect better and obtain appropriate conditions before entering the laboratory and testing on cells. In fact, we will have a virtual lab that will save time and reduce the cost. Therefore, in this study, numerical evaluations of fluid flow and cell tracing in the microfluidic chip were performed and validated with an experimental assessment of cell lines. The appropriate conditions of cell injection that have been found through simulation were used to prepare the chondrocyte-imprinted substrate. After chondrogenic differentiation of stem cells, in vitro assessments such as immunocytotoxicity and real-time PCR were done.

## Material and methods

### Microfluidic chip design

**Figure 1:**
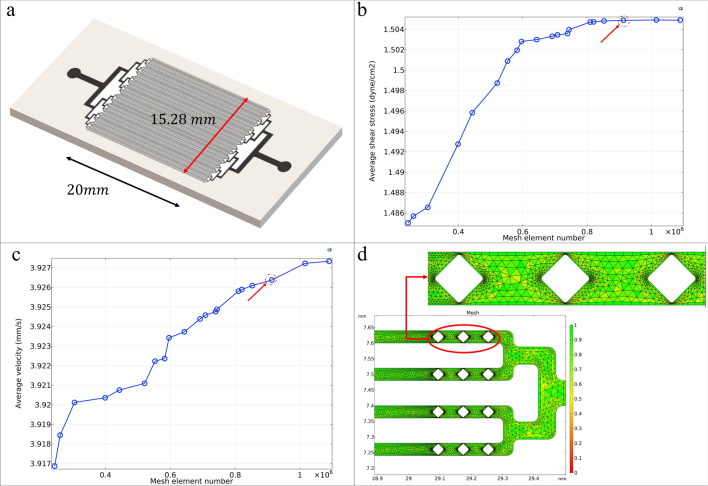
(**a**) Design of the microfluidic chip with 128 40 μm microchannels. (**b**) Convergence study for average shear stress in the microfluidic chip. (**c**) Convergence study for average velocity microfluidic chip. (**d**) Mesh plot around terminal microposts at the end of each microchannel. The color bar shows the mesh quality.

The location of the template cells should be monitored to improve the efficiency of the imprinting process, so a set of 128 microchannels (20 mm length and 50 μm depth) with a width of 25 μm and 40 μm comparable to that of template cells have been considered (Fig. 1a and FigS1.a). Approximately 2 × 10^6^ cells (with an average diameter of 8 μm) can be placed in regular and parallel lines in a microfluidic chip with 40 μm microchannels. At the end of each microchannel, three diamond-shaped microposts were considered to inhibit cells’ removal from their ends during cell injection. These microposts limited the available space’s width to 2 μm per side of the microchannel for moving cells while providing the cell culture medium exchange (Fig. 1d). The input channel’s width is 0.6 mm and 0.96 mm in the chip with 25 μm and 40 μm microchannels, respectively.

### Computational fluid dynamics analysis

Computational fluid dynamics (CFD) modeling is a useful technique that has been used in the field of microscale cell culture. It allows a deeper understanding of the function of the hydrodynamic environment and the factors that regulate it. CFD is generally applied to chemical and mechanical engineering; recently, it is used to consider the effects of fluid flow on cell function and offers valuable insights into microfluidic cell culture chip design and optimization. Thus, before fabrication, microfluidic cell culture device designs and their respective flow rates and patterns can be theoretically evaluated and characterized. Further precise parameters such as fluid inlet velocities and channels dimension can also be varied to better predict their effect on shear stresses, thus optimizing cell growth conditions^31,39,52,53^.

#### Mathematical model

Navier–Stokes equations explain how the velocity, pressure, temperature, and density of a flowing fluid are associated and include the influence of viscosity on the flow^39,54,55^. The continuity and Navier–Stokes equations (Eqs. (1) and (2)) for three-dimensional incompressible Newtonian fluid flow are:

1 $$ \nabla .V = 0 $$

2 $$ \frac{\partial V}{{\partial t}} + V.\nabla V = - \frac{1}{\rho }\nabla P + \frac{\mu }{\rho }\nabla^{2} V $$

where *V* represents the velocity vector (m/s),$\rho$ is the fluid’s density (kg/m^3^), *P* represents the pressure (Pa), and $\mu$ is the fluid’s viscosity (kg/(m.s).

The Reynolds number (Eq. (3)) indicates the ratio of inertial forces to viscous forces that describes the fluid flow regime.

3 $$ Re = \frac{\rho Ul}{\mu } $$

where *U* represents the average velocity (m/s) and *l* is the characteristic length (m). As microfluidic cell culture chips are very small in size, the flow regime is typically laminar^39,56,57^. At very low velocities, inertial forces become very weak compared to viscous forces, and the Reynolds number is less than 1. So the inertia terms to the left of Eq. (2) can be ignored^57,58^. The Navier–Stokes equation is reduced to the Stokes equation in this regime called Stokes or creeping flow (Eq. (4)). Creeping flow is a kind of fluid flow in which the advective inertial forces are low compared to the viscous forces.

4 $$ \frac{\partial V}{{\partial t}} = - \frac{1}{\rho }\nabla P + \frac{\mu }{\rho }\nabla^{2} V $$

As the Reynolds numbers are almost small in the creeping flow regime, a particle rotation and the Magnus lift force exerted on it can be ignored, and the drag (resistance) force in Stokesian form can be used^59^. In the Stokes limit, the lift force, an inertia-induced force, reduces to zero^60^. The Stokes law for calculating the Stokes drag force is shown in Eq. (5), where *V*_*p*_ is the particle velocity, *V* is the fluid velocity, and d_p_ is the particle diameter^59^.

5 $$ F_{d} = 3\pi d_{p} \mu \left( {V - V_{p} } \right) $$

In this study, to better understand the microfluidic chip’s flow characteristics, a numerical simulation of the microchip was computed using the COMSOL Multiphysics software. According to our microfluidic chip application for cell culture, its small microchannels, and low velocities in them, for the simulation, a 2D creeping flow model based on the steady-state Navier–Stokes’ equation and the particle tracing model for fluid flow were used. Experimental results for the density and viscosity of conventional cell culture mediums have shown that the measured values do not differ much from the properties of water. So in this study, the culture medium was considered an incompressible, Newtonian fluid with 1000 kg/m^3^ density and 0.001 Pa s dynamic viscosity^52,54,61,62^.

The boundary conditions of inlet velocity and zero pressure were used at the inlet and outlet, respectively, and no-slip conditions were applied to all walls. Cells were released in random positions at the chip’s inlet with a coupling velocity from the creeping flow model. In the particle tracing model, drag force based on Stokes’ equation was applied. For the initial assessment, the injection flow rate of 2.12 ml/h for syringe pump (equivalent to 0.012291 m/s and 0.019666 m/s inlet velocities in the chip with 40 μm and 25 μm microchannels respectively) and 2 × 10^6^ cells in 170 μl of culture medium with an average diameter of 12 μm and normal distribution of particles were considered.

#### Mesh independence study

A convergence and mesh independence study was performed for various mesh sizes to investigate mesh element number effect on average shear stress and velocity in the whole chip surface (Fig. 1b,c and FigS1.b-c). The final mesh with minimum element qualities of 0.21 and 0.27 and average element qualities of 0.74 and 0.69 was selected for the chips with 40 μm and 25 μm microchannels, respectively (Fig. 1d and FigS1.d). As shown in the figures, the percentage error with the converged value can be ignored for the selected mesh. Therefore, the selected mesh provides the answer with high accuracy and appropriate calculation time. Finer mesh has been used in the areas around the microposts, which have increased velocities due to reduced passage width.

After the appropriate mesh selection, the fluid flow and cells tracing model inside the microfluidic chip were solved. Also, various parameters such as fluid inlet velocity, number of cells, and cells’ sizes were changed to obtain suitable laboratory conditions. First, the results of this study were verified by experimental evaluations on cell lines. The appropriate values for cell concentration per unit volume of cell culture medium and injection flow rate of syringe pump chosen based on simulation results were then used to prepare chondrocyte-imprinted substrate and future stem cells chondrogenic differentiation in a cell-imprinted-based integrated microfluidic device.

### Experimental analysis

#### Cell culture

All the experiments were approved by the ethics committee of the Pasteur Institute of Iran, and all methods were performed in accordance with the relevant guidelines and regulations. The study was carried out in compliance with the ARRIVE guidelines. In this study, HUVEC, L929, and SW1353 cell lines also isolated Adipose-derived stem cells (ADSCs) and chondrocytes from 6-month-old male New Zealand white rabbits were used. Stem cells and chondrocytes were isolated from sacrificed animals in other studies according to the protocols established at the National Cell Bank of Iran^2,20^. In short, anesthesia was induced by injecting ketamine (35 mg/kg) and xylazine (8 mg/kg) intramuscularly. Then to harvest samples, barbiturate (100 mg/kg) was injected intraperitoneally. Harvested samples of Hyaline cartilage were washed multiple times with cell culture medium, sliced, and added to the trypsin–EDTA solution (0.25%, Sigma, USA) and placed in the incubator (37 °C). After 30 min, the samples were digested overnight in collagenase type II solution (0.08 mg/ml, Sigma, USA) in the incubator (37 °C and 5% CO2). The chondrocytes have the spherical morphology of mature cells a short time after isolation, but they de-differentiate and gain a spindle-shaped morphology after cultivation in a cell culture plate and more extended incubation (~ 14 days); so we used freshly isolated chondrocytes in this study^2^.

For ADSCs isolation, the adipose tissue was collected from the rabbit interscapular region. First, it was put in the DMEM cell culture medium containing antibiotic/antimycotic solution (1%, Invitrogen, USA). After separation of connective tissues, blood vessels, and fragmentation, the fragments were washed with PBS solution containing antibiotic/antimycotic (1%, Invitrogen, USA). Afterwards, they were added to collagenase type I (0.02 mg/ml, Sigma, USA) and were kept for 1 h in the incubator at 37 °C. The cells were then centrifuged, washed, and transferred to the culture medium containing Dulbecco’s Modified Eagle’s Medium (DMEM, GIBCO, Scotland)/Ham’s F12 supplemented with 100 μg/ml streptomycin, 100 U/ml penicillin (Sigma, USA), and 10% fetal bovine serum (FBS, Seromed, Germany).

According to our previously published report^63^, ADSCs’ multi-potency was assessed in vitro for adipogenesis, osteogenesis, and chondrogenesis.

#### Microfluidic device fabrication

Basic photolithography accompanied by deep reactive-ion etching (DRIE) of silicon using an oxide mask was used for the master fabrication process. Afterwards, using soft lithography, the pattern was transferred to PDMS (Sylgard 184 Silicon Elastomer Kit, Dow Corning) with the 10:1 weight ratio of the base polymer to the curing agent according to the previously published report^64^.

#### Cell-imprinted-based integrated microfluidic device fabrication

First, a cell-imprinted substrate was prepared using a microfluidic chip. For this purpose, after sterilization by treatment in the autoclave, a microfluidic chip with channels side facing down was placed on a cell culture plate. The solution with the concentration of 6 × 10^6^ of template cells in 150 μl of culture medium was prepared and injected into the chip using a syringe pump with a flow rate of 50 μl/min. The cell injection was continued until all the microchannels were filled with the cells and gained the desired pattern. In order to ensure the filling of microchannels, the microfluidic chip was observed under a microscope during the injection.

The set was put inside the incubator for 7 h to enable the cells to adhere to the cell culture plate’s surface while obtaining the microfluidic chip pattern. After removing the microfluidic chip from the cell culture plate, the plate’s surface, which had the pattern of template cells, was washed with PBS. Then the adhered template cells were fixed by 4% glutaraldehyde solution for 1 h. After washing the fixed template cells with distilled water and, after drying, PDMS casting was done. In order to transfer the cell pattern to the PDMS curing process was carried out at 37 °C for 1 day. After completing the curing process, the silicone layer was peeled off from the cell culture plate. The cell-imprinted substrate was then washed with 1 M NaOH solution to remove the residues.

For the fabrication of a cell-imprinted-based integrated microfluidic device, a new microfluidic chip should be aligned on the cell-imprinted substrate and attached to it using argon plasma. As the microfluidic chip’s design is in parallel lines, and the cell-imprinted substrate pattern is the same as the microfluidic chip pattern, they can easily align under the microscope after argon plasma treatment. Then in order to ensure the bonding of the upper microfluidic chip and the bottom cell-imprinted substrate, the cell-imprinted-based integrated microfluidic device was placed on the 80 ºC hot plate for 1 h. This cell-imprinted-based integrated microfluidic device can be used for future cell culture for biomedical applications such as drug analysis on template cells or stem cell differentiation to template cells. After removing cells with trypsin injection, washing and sterilization, the cell-imprinted-based integrated microfluidic device can be used again.

#### Application of the cell-imprinted-based integrated microfluidic device in stem cell differentiation

A cell-imprinted-based integrated microfluidic device was fabricated based on chondrocyte as template cell according to the above procedure. It was then sterilized using autoclave treatment. There is a static and a dynamic stage for differentiation of ADSCs in the cell-imprinted-based integrated microfluidic device. In the static stage, the solution with the concentration of 3 × 10^6^ of ADSCs in 150 μl of culture medium was prepared and injected into the integrated microfluidic device using a syringe pump with a flow rate of 50 μl/min until the cells filled all the microchannels. In order to allow the attachment of cells to the surface of the cell-imprinted substrate, the integrated microfluidic device should be placed inside the incubator for 4 h.

A parallel network of 4 cell-imprinted-based integrated microfluidic devices was used to have more differentiated ADSCs to chondrocytes simultaneously. This network was connected to a 25 ml syringe full of cell culture medium. In a dynamic stage during 14 days of ADCSs differentiation to supply the cell culture medium dynamically, a syringe pump with a flow rate of 1 ml/day was connected to the network mentioned above, and this set was placed in the incubator for 14 days. There is no need to change the cell culture medium by an operator like traditional cell culture by this method.

Trypsin–EDTA treatment was done using an insulin syringe instead of pippets in the traditional digestion methods in cell culture plates to remove differentiated cells from inside the cell-imprinted-based integrated microfluidic device after 14 days.

#### Microscopy observations

Scanning electron microscopy (SEM) was used to characterize the cell-imprinted substrate and observe its structure and morphology.

The upper microfluidic chip was not bonded to the bottom cell-imprinted substrate for better and easier imaging for fluorescence microscopy. So, after ADSCs culture on a cell-imprinted substrate in a microfluidic chip, the microfluidic chip can be removed. For this purpose, to prevent leakage, two rigid plexiglass sheets were used to compress the upper microfluidic chip and the bottom cell-imprinted substrate, which were aligned under a microscope. Cultured cells on the cell imprinted substrate were then fixed for 20 min in paraformaldehyde (4%, Sigma, USA) before staining. Antibodies employed for staining are shown in Table 1.

**Table 1:** Employed antibodies for staining.

Product type	Description
Phalloidin, FITC conjugated	Staining of the actin filaments
Fluorescent AlexaFluor488 labeled wheat germ agglutinin (WGA)	Cell membrane visualization
Primary antibody	Rabbit Polyclonal Antibody Collagen II (LS-C354627)
Secondary antibody	Goat Anti-Rabbit IgG H&L (FITC) (ab6717)

After 5 days of ADSCs culturing on the cell-imprinted substrate, FITC conjugated phalloidin (Sigma, USA) was used for the actin filaments staining; also, samples were stained with fluorescent AlexaFluor488 labeled wheat germ agglutinin (WGAAlexaFluor488, Invitrogen, USA).

After 14 days of chondrogenic differentiation of cultured ADSCs on a cell-imprinted substrate, Alcian blue staining was done to evaluate the proteoglycan expression. A solution of 2.5% glutaraldehyde (diluted from 50% solution; Merck) was prepared in25 mM sodium acetate and 0.4 M MgCl_2,_ and to have 0.05% concentration, Alcian blue 8GX (Sigma, Germany) dissolved in the prepared solution. After Alcian blue staining, the samples were washed with a 3% acetic acid solution^65^.

In addition, after 14 days of chondrogenic differentiation of cultured ADSCs on a cell-imprinted substrate, immunofluorescence staining of collagen type II was done^5,20^. The samples were washed with ice-cold PBS two times and incubated for 10 min in 0.25% Triton X-100 to permeabilize the cell membrane. Then, they were washed with PBS three times for 5 min each. Afterwards, they were incubated with 1% BSA for 30 min to block the secondary antibody reaction as the additional background color and subsequently incubated with the primary antibody (1: 100 dilution with PBS) for 1 h at room temperature, followed by washing with PBS three times for 5 min each. Cells were then incubated in the dark with secondary antibody (1:150 dilution with PBS) for 1 h at room temperature, followed by washing three times for 5 min each in the dark. After four washes in the dark, DAPI (Invitrogen, USA) was added and removed immediately. Then PBS was poured onto the samples, and they were evaluated using a fluorescent microscope (Labomed tcs400).

#### Gene expression analysis

In order to determine the expression of Aggrecan, Collagen I, Collagen II, and Sox9 genes, the Real-time PCR assay by StepOne instrument was used (Applied Biosystems, USA), and to design forward and reverse primers, the sequences of target genes were obtained from the NCBI database (Table 2). In this study, undifferentiated (normal) ADSCs and differentiated ADSCs (on the traditional cell-imprinted substrate and in a cell-imprinted-based integrated microfluidic device) were considered as control and test groups, respectively. According to the manufacturer’s instruction, a blood/Cultured cell total RNA mini kit (Yekta Tajhiz Azma, Iran) was used for total RNA isolation from samples after 14 days. Recombinant DNase I (TaKaRa, Japan) was used for removing Genomic DNA. Then the PrimeScript RT Reagent Kit (TaKaRa, Japan) was used for reverse transcription of total RNAs to produce single-strand cDNA. Finally, SYBR Premix Ex Taq II (Takara, Japan) was used for the Real-time PCR analysis with GAPDH as an endogenous control. The comparative Ct method was used for analyzing each gene expression quantitatively. Each target gene Ct value was normalized to their respected GAPDH.

**Table 2:** Primers sequences used in Real-time PCR ^a^.

Name of the corresponding protein		Sequence (5′ 3')	Length (bp)
GAPDH	Fw	5′-GGCACAGTCAAGGCAGAGAAC-3′	115
	Re	5′-CCACATACTCAGCACCAGCATC-3′	
Aggrecan	Fw	5′-CACCACGCCTTCTGCTTCC-3′	105
	Re	5′-TGTCACCATCCACTCCTCCAC-3′	
Collagen I	Fw	5′-GTCCTTCTGGTCCTCGTGGTC-3′	159
	Re	5′-CTTCGCCATCATCTCCGTTC-3′	
Collagen II	Fw	5′-GGAGCAGCAAGAGCAAGGAC-3′	151
	Re	5′-TGAGAGCCCTCGGTGGAC-3′	
Sox9	Fw	5′-GCTGGACTGGGAGTTGGAGAG-3′	179
	Re	5′-AAGGCGAATTGGAGAGGAGG-3′	

*Fw* forward, *Re* reverse.

## Results

### Computational fluid dynamics analysis

**Figure 2:**
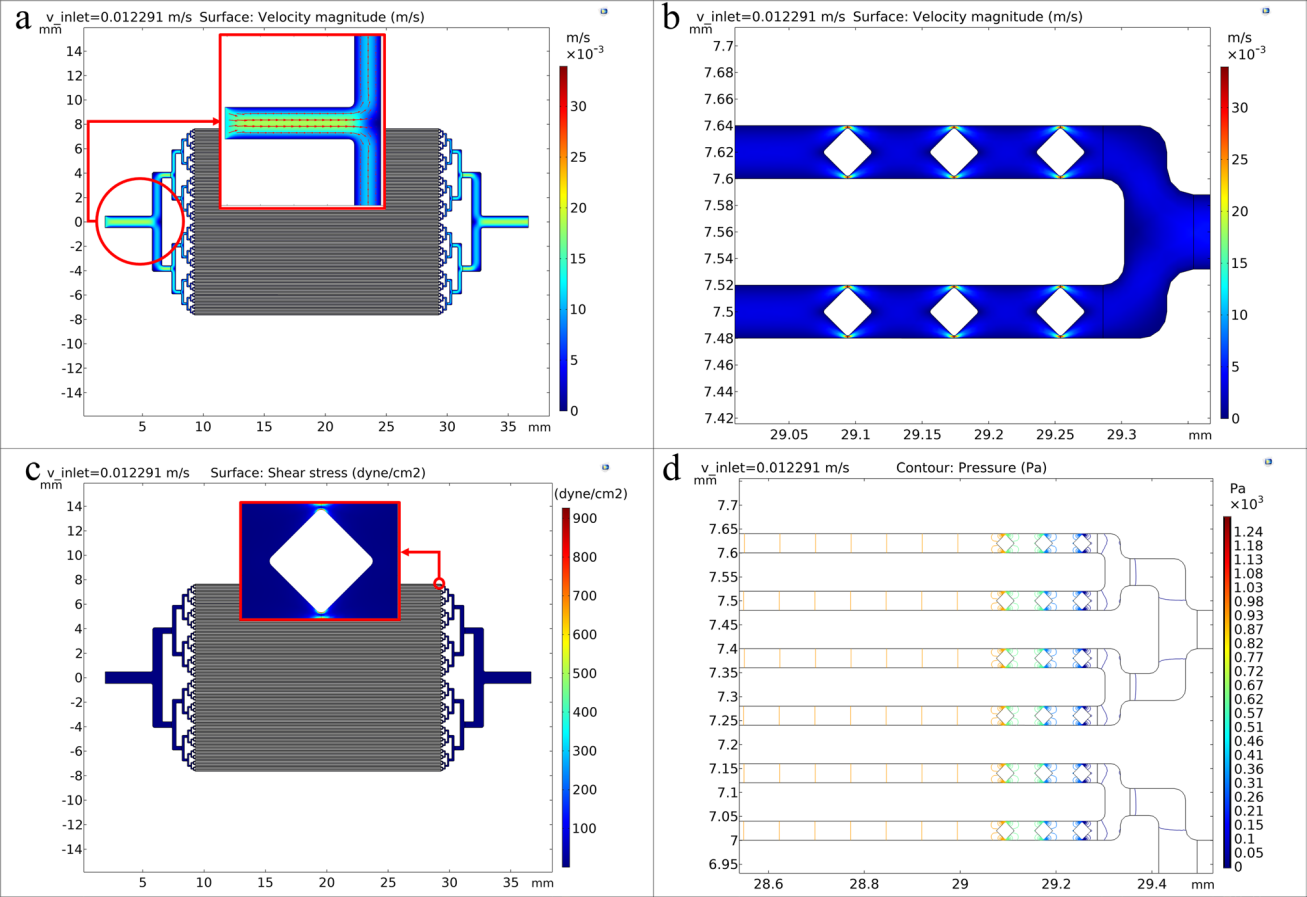
(**a**) Velocity profile in the microfluidic chip. (**b**) Velocity profile in the microfluidic chip around terminal microposts. (**c**) Shear stress profile in the microfluidic chip. The shear stress profile around a terminal micropost is zoomed. (**d**) Pressure contour in the microfluidic chip around terminal microposts. All data were obtained for the microfluidic chip with 40 μm microchannels.

As it was mentioned in Sect. “Computational fluid dynamics analysis.”, after the mesh convergence study and a proper mesh selection, simulation of the chip with 40 μm microchannels with the inlet injection velocity of 0.012291 m/s and the concentration of 2 × 10^6^ cells in 170 µl cell culture medium was performed. As not all cells are the same size, a normal distribution of cell size with an average diameter of 12 μm (almost the same as SW1353 cell line) was considered in this simulation. Figure 2a shows the velocity profile in the microfluidic chip with 40 μm microchannels, and Fig. 2b shows the velocity profile around terminal microposts. As can be seen in the microfluidic chip, we have laminar flow. Around the terminal microposts, where the available space for fluid passage decreases, the velocity increases, and maximum velocity occurs in these 2 μm free spaces between the chip and microposts walls.

When high shear stress exists, a cell membrane ruptures, and this phenomenon is called cell disruption. It is the principal physical cause of the death of cells. Born et al. studied the damage to suspended cells due to shear stress and reported the shear stress range of 2000–7000 dyne/cm^2^ for cell disruption in laminar flow. Figure 2c shows the shear stress profile in the microfluidic chip with 40 μm microchannels and around terminal microposts. Figure 2d shows the pressure contour around terminal microposts. As shown in the microfluidic chip, the shear stress applied to the cells is almost the same along the microchannels. Around the terminal microposts, where the available space for fluid passage decreases, the shear stress increases, and maximum shear stress occurs in these 2 μm free spaces between the chip and microposts walls. The surface average shear stress in the microfluidic chip is 1.504 dyne/cm^2^. This number is below the physiological shear stress of 10 dyne/cm^2^ experienced by vascular endothelial cells^66^.

As mentioned, when the cell-imprinted substrate is made, the cells are injected into the chip in a relatively short time. Then the chip is separated from the syringe pump and placed inside the incubator. For stem cell differentiation application of the cell-imprinted-based integrated microfluidic device, the stem cells are injected into the integrated microfluidic device and differentiate into chondrocytes in the incubator during the dynamics injection of the culture medium using a syringe pump for about 14 days. So, the stress applied to the cells inside the microfluidic chip should be consistent with the in vivo conditions at this stage. Therefore, fluid flow simulations were performed for lower flow rates of the culture medium in the dynamic stage. For a flow rate of 250 μl/day of culture medium per chip, the surface average shear stress in the microfluidic chip was 7.34 × 10^–3^ dyne/cm^2^. In previous experiments, the interstitial fluid level in the intra-articular cartilage surface and various articular surface layers was in the range of 10^–5^ to 10^–2^ dyne/cm^2^. So, the average shear stress applied in this work is consistent with the cartilage space’s interstitial fluid level.

Mechanotransduction is the response of cells to physical forces, and conversion of them into biochemical signals and mechanically activated (MA) ion channels act as physical force sensors^67^.Piezo1 and transient receptor potential vanilloid channels (TRPV4) are expressed in mechanosensitive cells such as endothelial cells and chondrocytes. TRPV4 and Piezo1 are non-selective calcium-permeable ion channels that can be activated by mechanical stimuli such as compression, hydrostatic pressure, or fluid flow (i.e., shear stress). TRPV4 is thought to operate as a signal transducer that responds to shear stress^68–72^. Shear stress regulates intracellular calcium [Ca^2+^]_i_ in endothelial cells. Recently shear stresses at physiological (≤ 5 dyne/cm^2^) and pathological levels were used to determine the amount and duration of shear stress forces that influenced [Ca^2+^]_i_ in HUVECs. A force of 12 dyne/cm^2^ applied for 1 min resulted in a sustained increase in [Ca^2+^]_i_, while the same force applied for 1 or 5 s, or lower shear stress (4 dyne/cm^2^) for 1 min was not enough to induce a sustained increase in [Ca^2+^]_i_ and resulted in just a transient increase^70^.

As it was mentioned, mechanical stimulation can stimulate both Piezo1 and TRPV4 in chondrocytes, and mechanotransduction is critical for articular cartilage health and normal tissue remodeling. To preserve a sufficient amount of cartilage, mechanotransduction in chondrocytes enables them to change the composition of the extracellular matrix (ECM). The cartilage can be damaged if mechanotransduction pathways are disturbed, leading to osteoarthritis and other joint diseases^73,74^. TRPV4 is abundantly expressed in articular chondrocytes, and loss of TRPV4 activity leads to osteoarthritis and joint arthropathy. The mechanically sensitive ion channel (MSC) mechanism in chondrocytes is critically important in clinical practice. Under abnormal mechanical load or excessive mechanosensitive ion channel activation, which can accelerate chondrocyte and ECM deterioration, the matrix stiffness changes, and mechanical signals are sent to the chondrocytes, activating TRPV4 and inducing a significant amount of calcium influx. Excessive activation of the Piezo1 ion channel, which can sense mechanical stimulus too, can cause apoptosis and mechanical injury. When a high-strain mechanical load is transferred to chondrocytes, which may cause injury, Piezo1 channel protein is activated, whereas low-strain physiologic loading is mediated by TRPV4^73–78^. One of the earliest responses of chondrocytes to physical stimulus is [Ca^2+^]_i_ signaling. Compression of the ECM will compress the interstitial fluid out of the cartilage, causing fluid flow or shear stress on the cell membrane in principle. Fluid flow velocity in the loaded cartilage is very slow due to the low permeability of the ECM, and the resultant fluid shear stress on the cell membrane is insignificant in contrast to studies of fluid flow-induced calcium signaling (5–40 dynes/cm^2^). So fluid flow could be a minor stimulus responsible for the [Ca^2+^]_i_ responses of in situ chondrocytes^68^. As it was mentioned, the fluid flow velocity in this work was so slow too. According to simulation results, the average shear stress applied in this work was 7.34 × 10^–3^ dyne/cm^2^, consistent with the cartilage space’s interstitial fluid level. Hence, it is not sufficient to be a major stimulus for activating mechanosensitive ion channels such as TRPV4 and Piezo1 in response to shear stress.

**Figure 3:**
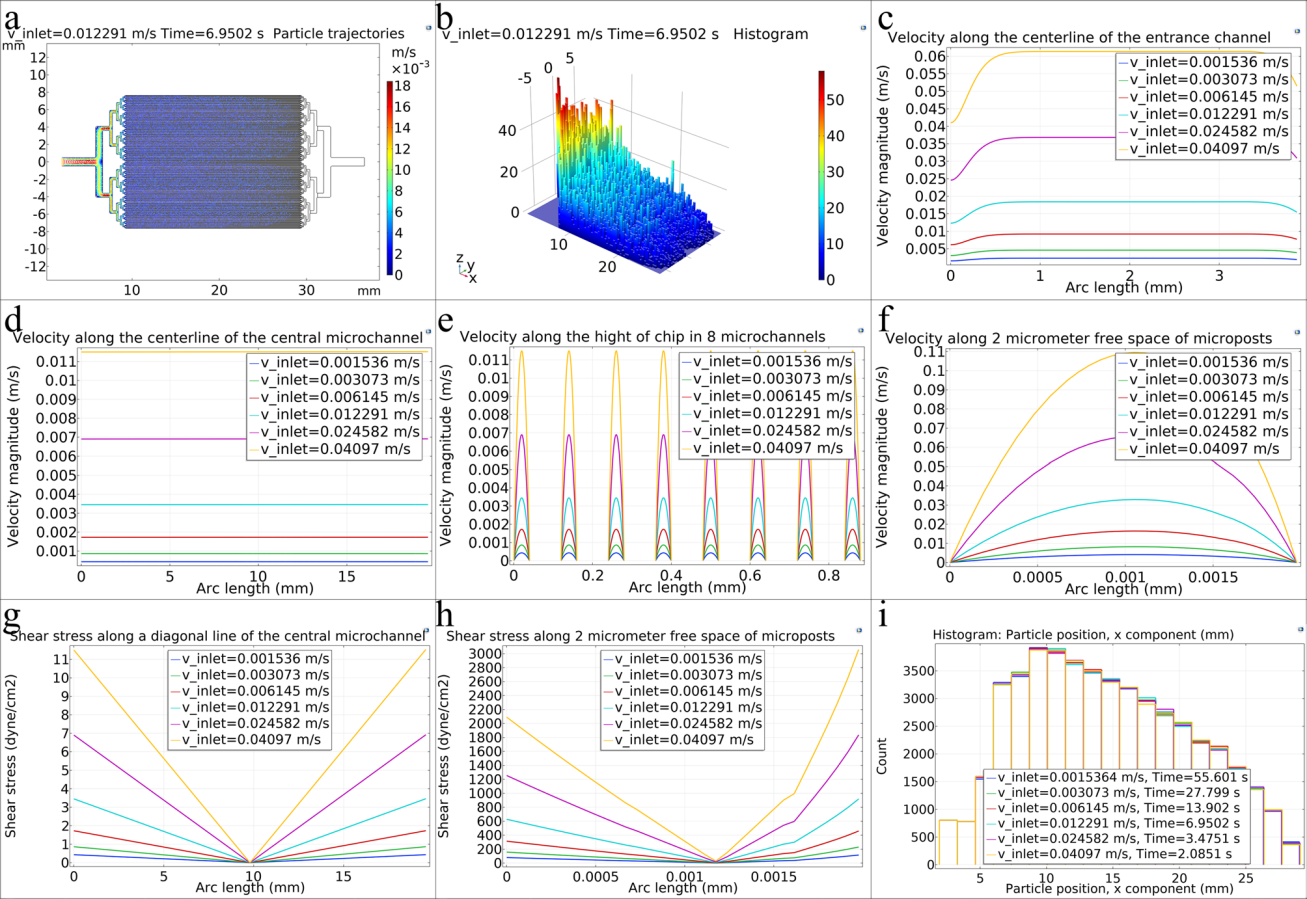
(**a**) Cell trajectories in the microfluidic chip when the first cells reach the terminal microposts. The cells’ color indicates their velocity (m/s). A scale factor of 4.9 is used for cell radius for better demonstration. (**b**) Histogram plot of cells distribution along the microfluidic chip when the first cells reach the terminal microposts. (**c**) Velocity changes along the input channel’s centerline. (**d**) Velocity changes along a cut line on the microfluidic chip’s central microchannel centerline (before reaching the central microchannel’s terminal microposts). (**e**) Velocity changes along the microfluidic chip’s height in eight consecutive microchannels. (**f**) Velocity changes in the direction of 2 μm vertical free distance between the micropost and the channel wall. (**g**) Shear stress changes along a diagonal cut line from the central microchannel’s bottom-left point to its top-right point (before reaching the central microchannel’s terminal microposts). (**h**) Shear stress changes in the direction of 2 μm vertical free distance between the micropost and the channel wall. (**i**) Histogram of cell position along the microfluidic chip. All data were obtained for the microfluidic chip with 40 μm microchannels, and (**c**–**i**) were obtained for different inlet velocities in the microfluidic chip.

Figure 3a shows the cell trajectories throughout the microfluidic chip when the first cells reach the terminal microposts. The color of the cells indicates their velocity (m/s) in the chip. As can be seen, the particles’ velocity is approximately the same along all 40 μm microchannels. With the help of simulation, the time when the first cells reach the end of the microchannels can be obtained. As shown in Fig. 3a, since the microfluidic chip design is symmetrical about the x-axis, the particle distribution on the chip surface is also symmetrical. The movement of cells in the top, bottom, and two central microchannels are behind the other microchannels, and these microchannels are the last microchannels to be filled. The movement of cells in the microfluidic chip can be obtained as animation using simulation and can be used as a guide before laboratory experiments (Supplementary video 1). This video can also be compared and validated by experimental injection of cells in the laboratory. Figure 3b shows the histogram of particle distribution throughout the microfluidic chip when the first cells reach the terminal microposts. As can be seen, almost all microchannels have received a similar distribution of cells, and the number of cells in the inlet sections, which are larger, is higher than the 40 μm microchannels. Also, the number of cells decreases along the microfluidic chip from the inlet towards the outlet.

Next, we investigated the effect of cells’ injection flow rate (input velocity in simulation). Figure 3c shows the velocity changes along the centerline of the input channel for different inlet velocities in the microfluidic chip with 40 μm microchannels. As can be seen, the velocity slightly increases, reaches a constant value, and decreases again after reaching the flow’s partition in the next channels. Figure 3d shows velocity changes along a cut line on the microfluidic chip’s central microchannel centerline (before reaching the central channel’s terminal microposts) for different inlet velocities. Also, Fig. 3e shows velocity changes in the vertical direction of a microfluidic chip in eight consecutive microchannels for different inlet velocities. As can be seen, all eight microchannels have the same velocity profile, and this is true for all microchannels; and according to Fig. 3d, the velocity remains constant along microchannels. So, according to Fig. 3d,e, it can be concluded that all microchannels of the chip have almost the same velocity profile. Figure 3f shows velocity changes in the direction of a 2 μm vertical free distance between the micropost and the microchannel wall for different inlet velocities. As can be seen in this area, the velocity is higher than other areas, and it is in parabolic shape because, in contact with the walls, the velocity is zero. However, in the distance between the micropost and the microchannel wall, the velocity increased by a reduction in available passage space.

Figure 3g shows the shear stress changes along a diagonal cut line from the central microchannel’s bottom-left point to its top-right point (before reaching the central microchannel’s terminal microposts) for different inlet velocities in the microfluidic chip with 40 μm microchannels. As can be seen, the shear stress is higher near the walls and tends to almost zero as it approaches the microchannel’s midpoint. Also, the higher the input velocity, the higher the shear stress applied to the cells. Figure 3h shows shear stress changes along the 2 μm vertical free distance between the micropost and the microchannel wall for different inlet velocities. Shear stress increases near the micropost and microchannel walls. Figure 3i shows a histogram of cells’ positions along the microfluidic chip at different inlet velocities. As the changes in input velocity also change cells release time and the time it takes for the first cells to reach the end of the channels, in a similar time index, the change in velocity does not affect the location and number of cells per location much. The point to be noted is that although the shear stress applied to the cells decreases as the inlet velocity decreases, the microchannels’ time to fill increases. This means that cells are out of the incubator for a longer time, and the possibility of settling the cells inside the syringe increases, which will cause an error in the experimental results.

**Figure 4:**
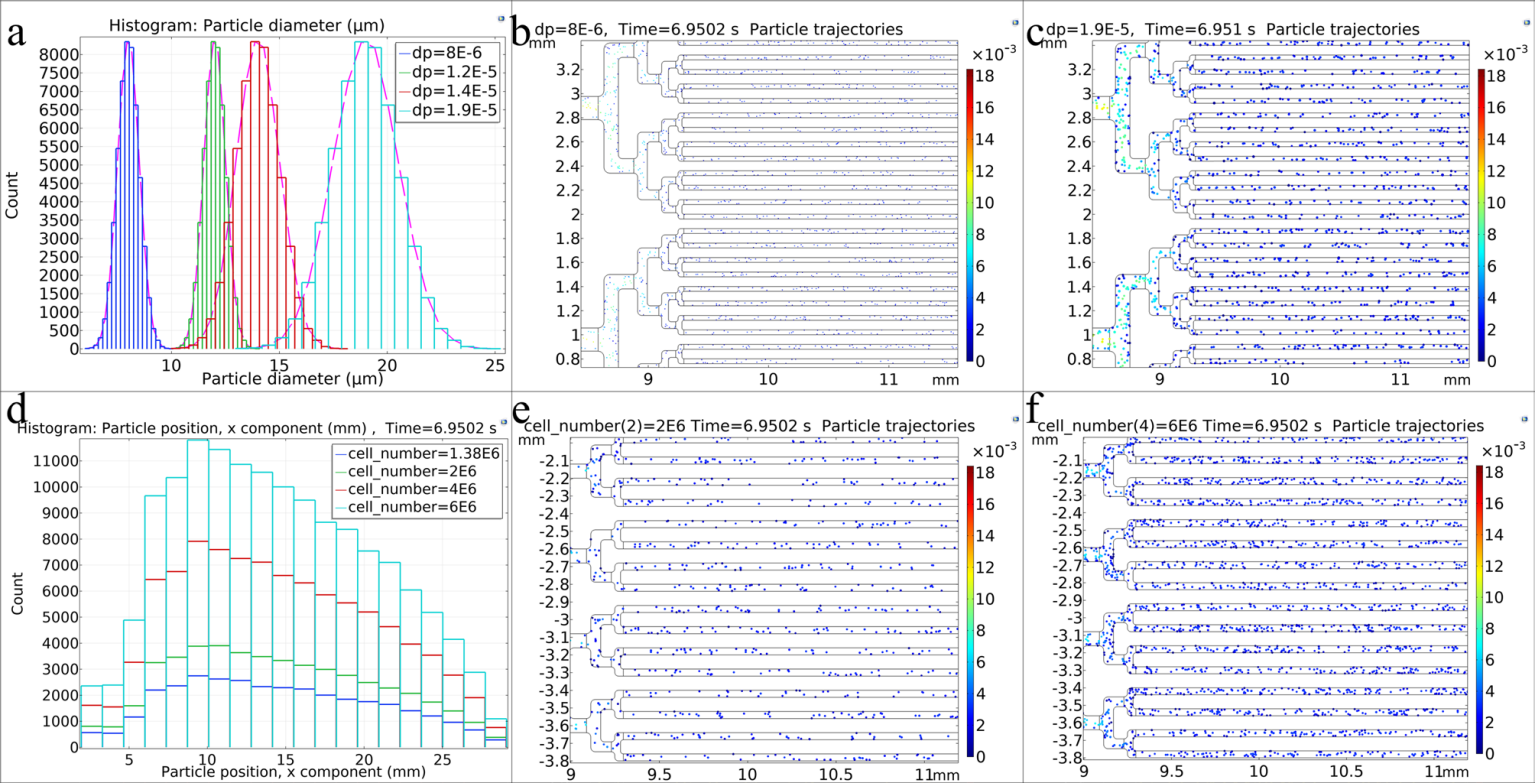
(**a**) Normal distribution of cell size for cells with average diameters of 8, 12, 14, and 19 μm in the microfluidic chip. (**b**) 8 μm diameter cell trajectories in the microfluidic chip. (**c**) 19 μm cell trajectories in the microfluidic chip. (**d**) Histogram of cell position along the microfluidic chip for different cell numbers in a constant volume of cell culture medium. (**e**) Cell trajectories in the microfluidic chip for a cell concentration of 2 × 10^6^ cell/170 μl cell culture medium. (**f**) Cell trajectories in the microfluidic chip for a cell concentration of 6 × 10^6^ cell/170 μl cell culture medium. All data were obtained for the microfluidic chip with 40 μm microchannels.

In addition, to study the effect of input velocity changes, similar to the above simulations, which were performed for cells with an average diameter of 12 μm in normal size distribution (Fig. 4a), other simulations were performed for cells with different diameters. Thus, the inlet velocity and the number of cells in 170 μl cell culture medium were fixed at 0.012291 m/s and 2 × 10^6^ respectively, but the cell diameter and cell size distribution were almost similar to freshly isolated chondrocytes, L9292 and HUVEC cell lines (average diameters of 8, 14 and 19 μm respectively). The particle size distribution profiles intended for these cells are shown in Fig. 4a. As can be seen, a wider particle size distribution is considered for larger cells, according to laboratory observations.

Figure 4b,c show cell trajectories in the microfluidic chip with 40 μm microchannels for two different average diameters of 8 μm and 19 μm. As can be seen, for the same concentration of 2 × 10^6^ cells in 170 μl cell culture medium and the same time index, the number of cells with 8 μm diameter is not enough to fill the microfluidic chip with 40 μm microchannels, so for smaller cells, the chip with smaller microchannels, and more of these cells in a constant volume of cell culture medium (which increase the injected cells concentration) should be used.

Next, the velocity (0.012291 m/s) and the diameter of the cells (12 μm) were considered constant as the initial simulation. However, the effect of increasing the number of injected cells in 170 μl volume of cell culture medium (increasing injected cells concentration) was investigated. Figure 4d shows the histogram of the cell’s position along the microfluidic chip with different numbers of cells in the constant volume of the injected culture medium. As can be seen, as the input cell concentration increases, the number of cells increases at the same spatial distance, resulting in a more orderly cell pattern. Figure 4e,f also show cell trajectories in the microfluidic chip with 40 μm microchannels for two different cells number in a constant volume of cell culture medium when the first cells reach the terminal microposts. As it can be seen, higher cell concentration leads to more cell-loaded microchannels.

All the above simulations were performed for microfluidic chips with 25 μm microchannels. Similar results were obtained for changes routine in terms of input velocity, diameter, and the number of cells. It is noteworthy that for cells with the same diameter, same cell number per unit volume, and the same injection flow rate of the syringe pump, the effect of reducing the width of the microchannel was on the values of velocity, shear stress, and the number of cells and time required to fill the microchannels (FigS2).

Tables 3 and 4 show a comparison of the surface average and the surface maximum along the microfluidic chip with 40 μm and 25 μm microchannels for shear stress and velocity. The rows are for the same injection flow rate of the syringe pump in both chips. Comparing the shear stress and velocity results for two chips with different microchannel diameters but with the same cell injection conditions shows that the surface maximum values ​​for both chips are approximately the same but increase for the surface average of ​​the chip with smaller microchannels. Due to the similarity of the syringe pump flow rate and identical geometry of terminal microposts in both chips, unlike their different diameters, the surface maximum values that occur at 2 μm free spaces between the chip and microposts walls are almost the same for both chips. However, the values increase in the smaller diameter chip for the surface average because the velocity increases with decreasing cross-sectional area. Also, since the microchannels are smaller in the 25 μm microfluidic chip, the number of cells along the chip length will decrease in almost identical locations (compare Fig. 3b and FigS2.f).

**Table 3:** Surface average of shear stress and velocity for two microfluidic chips with 40 μm and 25 μm microchannels.

Surface average					
40 μm			25 μm		
v_inlet (m/s)	Shear stress (dyne/cm^2^)	Velocity magnitude (m/s)	v_inlet (m/s)	Shear stress (dyne/cm^2^)	Velocity magnitude (m/s)
0.001536	0.18811	4.91E-04	0.002458	0.49106	7.27E-04
0.003073	0.37625	9.82E-04	0.004916	0.98211	0.0014538
0.006145	0.75238	0.001963	0.009833	1.9644	0.0029079
0.012291	1.5049	0.0039264	0.019666	3.9288	0.0058158
0.024582	3.0098	0.0078528	0.039331	7.8575	0.011631
0.04097	5.0163	0.013088	0.065552	13.096	0.019386

**Table 4:** Surface maximum of shear stress and velocity for two microfluidic chips with 40 μm and 25 μm microchannels.

Surface maximum					
40 μm			25 μm		
v_inlet (m/s)	Shear stress (dyne/cm^2^)	Velocity magnitude (m/s)	v_inlet (m/s)	Shear stress (dyne/cm^2^)	Velocity magnitude (m/s)
0.001536	115.79	0.0042421	0.002458	115.9	0.0041847
0.003073	231.59	0.0084848	0.004916	231.81	0.0083694
0.006145	463.1	0.016967	0.009833	463.67	0.01674
0.012291	926.28	0.033936	0.019666	927.33	0.033481
0.024582	1852.6	0.067873	0.039331	1854.6	0.06696
0.04097	3087.6	0.11312	0.065552	3091	0.1116

Therefore, considering the application of future cell culture in the cell-imprinted-based integrated microfluidic device, more differentiated stem cells are needed to place on scaffolds and transplant in the animal’s body; it is better to use a microfluidic chip with 40 μm microchannels, which has a higher capacity. Another point to note is that it takes less time for the first cells to reach the terminal microposts in a chip with smaller microchannels, and it should be noted that this short injection time is more difficult to control, and prolonging the injection time causes excessive cell accumulation. It affects their adhesion and may push cells out of the microchannels’ outlet by applying more pressure.

In this part, by simulation, the parameters which affect the experiment were investigated. Therefore, for cell-imprinted substrate preparation, in addition to selecting the appropriate inlet velocity that does not exert too much shear stress on the cells and does not allow the cells to be out of the incubator for a long time during injection so as not to damage the cells and cause the cells to settle inside the insulin syringe, the sufficient concentration of injected cells should be selected so that we can have a regular pattern of cells in parallel lines. Also, the injection flow rate of the dynamic stage cell culture medium can be selected so that applied shear stress in the integrated microfluidic device is consistent with the cartilage space’s interstitial fluid level. Simulation helps us get an overview of experimental conditions before entering the lab without wasting materials and time.

### Validation of numerical analysis with experimental analysis

The SW1353 cell line with an average diameter of 12 μm and a concentration of 1,380,000 cells in 170 μl cell culture medium in a microfluidic chip with 40 μm microchannels and a syringe pump flow rate of 2.12 ml/h were used to validate the simulation results. For this purpose, after preparing the cell injection conditions using a light microscope and a camera, a video of the movement of cells within fluid flow at a frame rate of 30 frames per second was recorded. To validate the simulation, we applied exactly the same conditions in the simulation and prepared an animation of the cell trajectories at the same frame rate. The chip’s position under the microscope was tried to be exactly the same as the part of the chip in the simulation from which the animation was prepared. Then both videos were put beside each other for comparison (Supplementary video 2).

Comparing simulation and experimental videos visually, we concluded that the simulation accurately predicted the movement of cells in the microfluidic chip and that the cell movements differed in velocity in a few hundredths of a second.

Techniques of velocimetry are commonly used in a variety of applications. In particular, particle image velocimetry (PIV) is a simple but often expensive technique to describe fluid flow fields. A high-speed digital camera is used in the PIV method to take consecutive images of the illuminated area in the fluid, which consists of particles that have been injected with a proper concentration to determine their position. Then, with the help of a numerical algorithm, the amount of particle displacement is determined by comparing these two images so the flow velocity component can be measured^79^. As fluid velocity is a parameter that affects the shear stress on cells, in this study, in addition to visually comparing simulation and experimental results, an image velocimetry method was used to compare the results quantitatively. Here supplementary video 2 was converted into consecutive images. Since the time interval between images is known, the velocity of the cells can be calculated by measuring their displacement along the microchannels and dividing it by time. Also, since the dimensions of the microchannels are known (40 μm), the images can be calibrated to get the actual amount of displacement. This method was performed for different time intervals and some of the cells. The average of cells’ velocity in the experimental video was compared with the average of cells’ velocity in the simulation. With this image velocimetry method, the average of cells’ velocity was 0.00371 m/s in the experimental and 0.00398 m/s in the numerical evaluation. As a result, the calculated percentage error was equal to 6.8%, which indicates that the numerical evaluation predicted our experimental results with a good approximation. This slight difference in simulation and experimental results may be due to the following:

1. When making a microfluidic chip, after several times of molding and removing the PDMS layer from the silicon wafer, some of the microposts may not form properly. They may remain inside the silicon wafer, causing some microchannels to miss some of their terminal microposts. So the resistance against the fluid flow and passing cells in some microchannels may be decreased. Compared with the ideal simulation of the same microchannels, this can cause some differences in experimental.
2. In experimental, cell mixing (by pipette up/down) may not be performed well, and the cell concentration inside the insulin syringe may not be uniform, and some cells may make aggregates.
3. There might be an error in cell counting, and the cell number might differ from what was counted by the hemocytometer slide and trypan blue staining (human error).
4. The syringe pump’s flow rate used to inject the cell into the microfluidic chip in the laboratory may not be exactly the set value.

Despite the above reasons, the simulation results agree with the experimental results, which shows the power of computational methods. In a virtual laboratory, suitable conditions for experimental studies can be obtained without wasting time and money in a real laboratory.

### Microscopy observations

**Figure 5:**
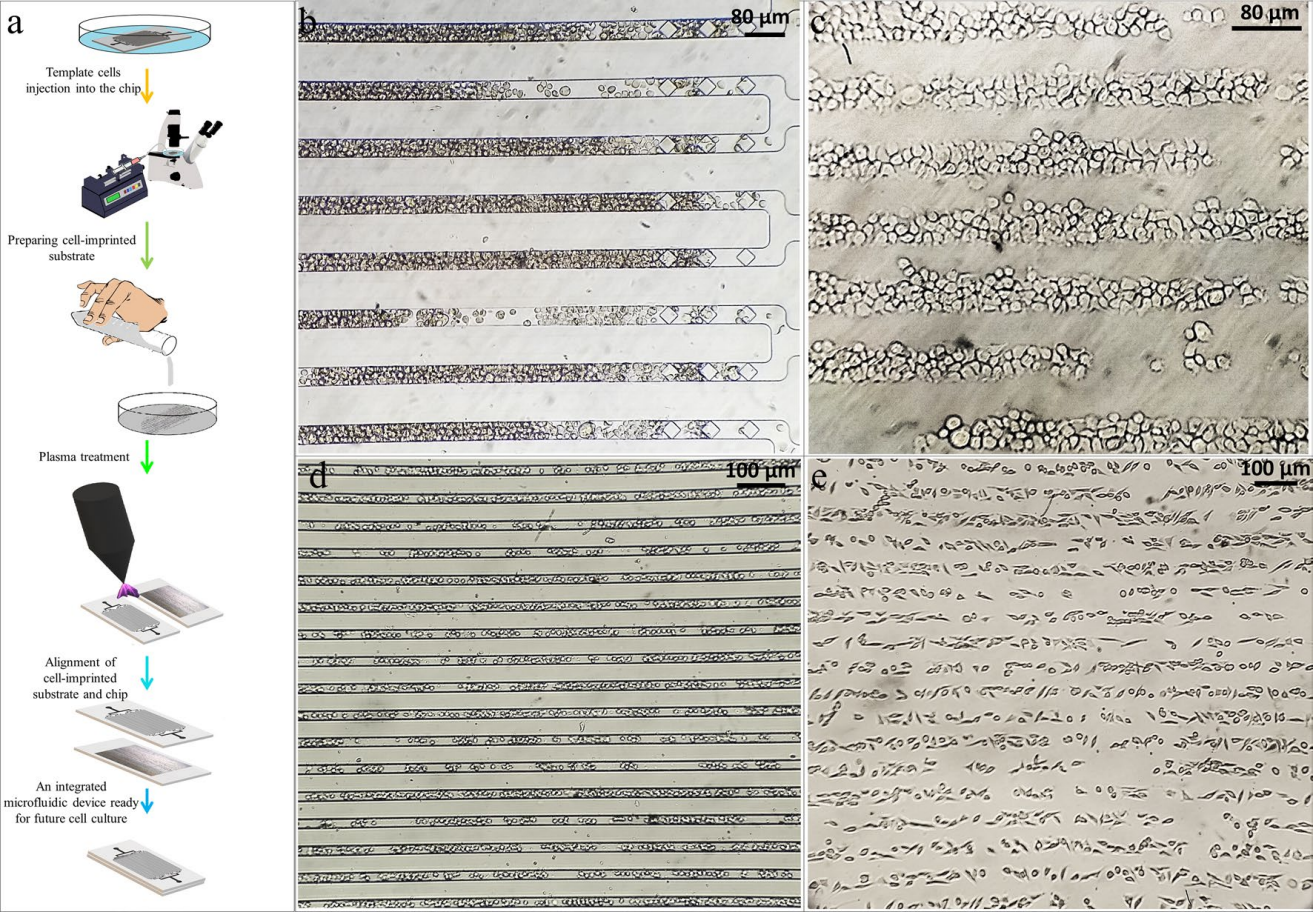
(**a**) The schematic of the cell-imprinted-based integrated microfluidic device fabrication procedure. (**b**) The function of the microfluidic chip in trapping the HUVEC cell line in an area around terminal microposts of 40 µm microchannels of the microfluidic chip, which is almost filled with cells after injection with a syringe pump. (**c**) The regular pattern of HUVEC cell lines transferred to the cell-culture plate. (**d**) 25 µm microchannels of the microfluidic chip are almost filled with L929 cell line after injection with a syringe pump. (**e**) The regular pattern of L929 cell lines transferred to the cell-culture plate.

Figure 5a shows the schematic of the cell-imprinted-based integrated microfluidic device fabrication procedure. Before experimenting with chondrocytes as the main cells in this study, which needs to be isolated from the rabbit’s cartilage, the microfluidic chip’s functionality in trapping cells and creating a regular pattern the same as the microfluidic chip geometry was evaluated for HUVEC and L929 cell lines.

The function of the microfluidic chip in trapping the HUVEC cell line and creating a pattern consistent with the geometry of the microchannels is shown in Fig. 5b, which shows an area around terminal microposts of 40 µm microchannels of the microfluidic chip after injecting the HUVEC cell line with a syringe pump. As it can be seen, terminal microposts at the end of each microchannel have almost well prevented cells from passing through the microposts and leaving the microfluidic chip. In addition, the microchannels were almost filled with cells in regular controlled places, and this pattern transferred to the cell-culture plate and looks like regular paving (Fig. 5c). FigS3.a also shows 40 µm microchannels of the microfluidic chip almost filled with HUVECs in an area away from terminal microposts. The same results are also shown in Fig. 5d for the L929 cell line in 25 µm microchannels of the microfluidic chip after injection and their regular pattern transferred to the cell-culture plate (Fig. 5e).

As it was mentioned, it was proved that instead of cell culture in conventional rigid polystyrene plates, cell-imprinted substrates based on their topography provide conditions similar to those of cells’ natural growth environment. In traditional imprinting methods, the placement of cells was random, and the probability of the cells to place exactly on the cell-imprinted topography was low.

In this study, by applying a microfluidic chip, the topography of template cells regularly transfers to the cell culture plate. So, a regular cell-imprinted substrate can be made after mold casting on these regular cellular topographies by PDMS. These regular cell-imprinted substrates can provide platforms for anti-cancer drug analysis in future studies by culturing the same cell line as the template cell line that has been used for cell imprinting.

**Figure 6:**
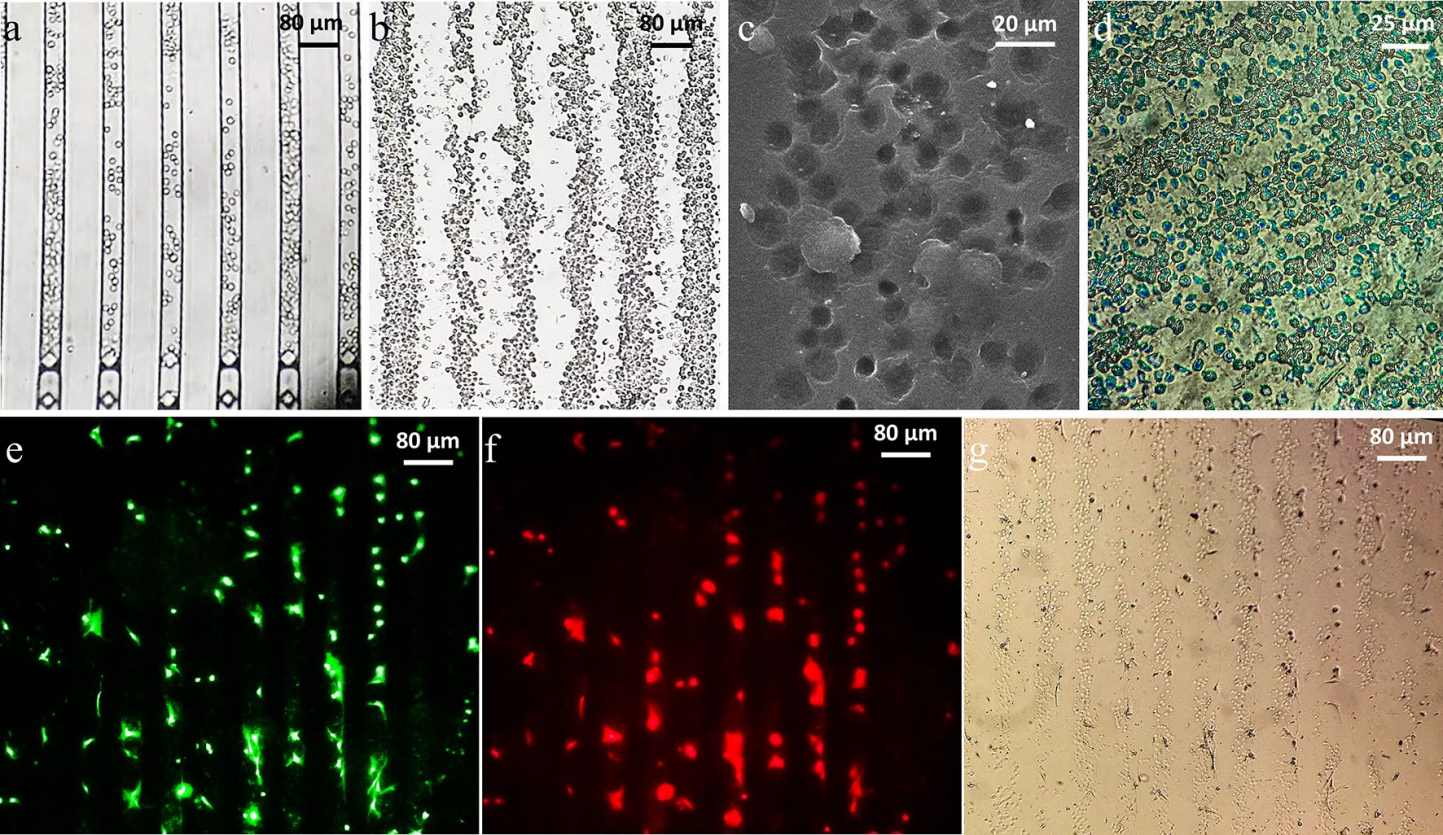
(**a**) The function of the microfluidic chip in trapping chondrocytes in an area around terminal microposts of 40 µm microchannels of the microfluidic chip, which is almost filled with chondrocytes. (**b**) The regular pattern of chondrocytes transferred to the cell-culture plate. (**c**) The chondrocyte-imprinted substrate SEM image was prepared by a microfluidic chip with 40 µm microchannels. (**d**) Stained ADSCs cultured in a 25 µm microfluidic chip on the cell-imprinted substrate with alcian blue. (**e**) Phalloidin staining of actins in cultured ADSCs in a microfluidic chip on the cell-imprinted substrate. (**f**) Immunostaining of ADSCs cultured in a microfluidic chip on the cell-imprinted substrate with fluorescent AlexaFluor488 labeled wheat germ agglutinin (WGA). (**g**) Optical microscopy of ADSCs cultured in a microfluidic chip on the cell-imprinted substrate.

After evaluating our microfluidic chip’s functionality with test cells (HUVEC and L929 cell lines), experiments were performed with chondrocytes, and they were injected into the microfluidic chip with 40 µm microchannels. The function of the microfluidic chip in trapping the chondrocytes and creating a pattern consistent with the geometry of the microchannels is shown in Fig. 6a, which shows the microchannels were almost filled with chondrocytes in regular controlled places and this pattern transferred to the cell-culture plate (Fig. 6b). Also, 40 µm microchannels of the microfluidic chip almost filled with chondrocytes in an area away from the terminal microposts are shown in FigS3.b. The SEM image of one line of the regular chondrocytes’ pattern on the cell-imprinted substrate is shown in Fig. 6c. As can be seen, chondrocytes’ topography in a regular arrangement similar to the microfluidic chip’s parallel microchannels was transferred to the PDMS replica by mold casting. Also, proteoglycan’s presence secreted by differentiated ADSCs on the cell-imprinted substrate surface in the microfluidic chip was confirmed by Alcian blue staining (Fig. 6d).

Phalloidin staining, WGA staining, and optical microscopy of ADSCs cultured for 5 days on a chondrocyte-imprinted substrate in a microfluidic chip are shown in Fig. 6e–g, respectively. As it can be seen, just after 5 days, the spindle morphology of ADSCs was almost converted into chondrocyte’s spherical morphology. Also, ADSCs created a regular pattern like that of the microfluidic chip on parallel lines in the predicted locations on the regular cell-imprinted substrate.

As it was mentioned, uncertainty over whether ADSCs have been specifically positioned on the chondrocyte pattern on the cell-imprinted substrate is the key issue with the traditional imprinting methods. The location of the cells is completely unpredictable. In this study, based on the parallel lines in microfluidic chip design, a similar chondrocyte pattern is formed on the cell–imprinted substrate. In the cell-imprinted-based integrated microfluidic device fabrication procedure, the upper microfluidic chip and the chondrocyte-imprinted substrate can be easily aligned on their similar lines before the bonding stage. This approach is objective, controlled, and non-random, unlike previous imprinting techniques. ADSCs’ pathway is predicted in the cell-printed-based integrated microfluidic device to be precisely positioned on the chondrocyte-imprinted pattern. After 14 days of differentiation, ADSCs will get the chondrocyte phenotype.

**Figure 7:**
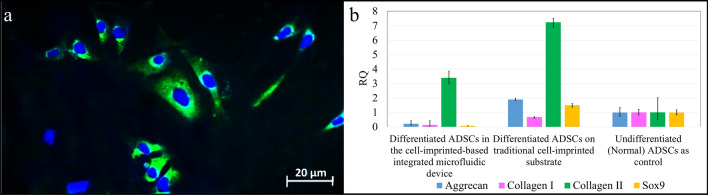
(**a**) Stained ADSCs in a 25 µm microfluidic chip on the cell-imprinted substrate with collagen II (green) and DAPI (blue) for the nucleus. (**b**) Quantitative real-time PCR analysis: gene expression profile of differentiated ADSCs in the cell-imprinted-based integrated microfluidic device and on the traditional cell-imprinted substrate and freshly isolated ADSCs which were grown on standard cell culture flasks as control; different genes (aggrecan, collagen I, collagen II, and sox9) are expressed relatively.

In addition, after 14 days, ADSCs cultured on the cell-imprinted substrate were stained with collagen II antibody, and cell nuclei were stained with DAPI (Fig. 7a). Also, evaluation of chondrogenic differentiation in 3 samples according to image processing with IMAGE J software showed collagen II (chondrocyte specific gene marker) relative expression of about 68%.

### Gene expression analysis

The cultured ADSCs gene expression analysis in the cell-imprinted-based integrated microfluidic device was compared with cultured ADSCs on the traditional cell-imprinted substrate (Fig. 7b). Undifferentiated ADSCs which were grown on standard cell culture flasks were considered as control. In ADSCs cultured in the cell-imprinted-based integrated microfluidic device compared with cultured ADSCs on the traditional cell-imprinted substrate, collagen II expression (a chondrocyte specific gene marker) is up-regulated while the expression of collagen I is significantly down-regulated.

As a criterion for comparison between chondrogenic differentiations in different methods, the ratio of collagen type II to collagen type I expressions was evaluated for cultured ADSCs in the cell-imprinted-based integrated microfluidic device and on the traditional cell-imprinted substrate. According to Fig. 7b, this ratio is significantly higher for differentiation of ADSCs in the cell-imprinted-based integrated microfluidic device (3.39/0.137 ~ 24.7) than the traditional cell-imprinted substrate (7.23/0.68 ~ 10.6).

## Discussion

Cell-imprinting technology is an innovative technique for directing stem cell fate by molding substrates from target cells. This method’s functionality has been proven in previous research for various cells for different applications such as drug analysis and stem cell differentiation^2,19–23,25,27,80^. The template cells are cultured and fixed on the cell culture plate’s surface in this method. Then by polydimethylsiloxane (PDMS, Sylgard 184) mold casting, the template cell cellular plasma membranes and nucleus topography is transferred to the cured PDMS layer, which can be used as a new cell-imprinted substrate for future cell culture.

The major issue with the conventional imprinting method is its poor efficacy because the cells’ location is random. Hence, the likelihood of the secondary cell being placed on the template’s cell-imprinted substrate is low. If a cell finds a pattern by chance, it would probably go inside the hole to form a new shape, but this is not a reproducible result, and there is no power to regulate cells’ migration into patterns according to previous experiments. The second weakness of previous cell culture approaches on a PDMS cell-imprinted substrate is synonymous with failures when a bubble is formed under the PDMS substrate. This PDMS substrate may be submerged in the culture medium, and the cells on the substrate of PDMS would remain without the medium. Also, there is the possibility of cells migrating from the PDMS substrate to the polystyrene culture plate, which can cause cell culture errors.

In this study, in order to eliminate the drawbacks of traditional imprinting methods, the placement of template cells was predicted by using a microfluidic chip, and imprinted cells got the same pattern as the parallel line of the microfluidic chip. In order to increase the efficiency of traditional imprinting methods using another microfluidic chip aligned on the cell-imprinted substrate, the culture of the secondary cells, which can be the same as template cell (while preserving normal cell activity) or stem cell (to be differentiated to the template cell) based on the biomedical application, is non-random and targeted. Also, the probability of secondary cells placement on the cell-imprinted substrate was increased. In addition, cell culture in this cell-imprinted-based integrated microfluidic device was dynamic using a syringe pump, and the culture medium passed continuously over the cells. In contrast with conventional cell culture methods, there was no need to change the cell culture medium and daily care by an operator, which increases the risk of error. Also, in the case of stem cell differentiation, a network of these cell-imprinted-based integrated microfluidic devices was connected to a syringe pump. It simultaneously supplied more differentiated ADSCs to chondrocytes without any chemical growth factors and only with a physical signal and improved stem cell differentiation efficiency, increasing the success of future cell transplantation procedures. This study’s cell-imprinted-based integrated microfluidic device can be washed after removing cells by trypsin–EDTA treatment and autoclaved again for further use. All of the above advantages led to a significant reduction in the final cost.

Phalloidin, WGA, and Alcian blue staining results showed that the spindle morphology of ADSCs cultured in the microfluidic device on the cell-imprinted substrate was converted into spherical morphology of chondrocyte by placement into the chondrocyte-imprinted topography. Collagen II and Alcian blue staining as a criterion of chondrogenic differentiation showed positive results. The gene expression analysis showed that ADSCs differentiation in the cell-imprinted-based integrated microfluidic device successfully increased collagen II expression and decreased collagen I expression than control, which was the undifferentiated ADSCs cultured on a cell culture flask. Compared to traditional imprinting, ADSCs differentiation was improved because the ratio of collagen II to collagen I expressions was 2.3 times higher for ADSCs differentiation in the cell-imprinted-based integrated microfluidic device than traditional cell-imprinting methods.

Previous experiments have shown that all cells with possible chondrogenic differentiation capability can be differentiated into chondrocytes on the traditional chondrocyte-printed substrate^20^. So in our chondrocyte-imprinted-based integrated microfluidic device, too, all cells with potential chondrogenic differentiation can be differentiated into chondrocytes.

This method, which is safe, cheap, reproducible, and well-controlled, can be used for all cell culture applications instead of cell culture plates in the laboratory or clinic. It can also be generalized to other adherent template cells or other cell transplantation methods, such as heart, skin, neuron, bone, etc. According to the final application, the template cell can be isolated from the tissue or chosen from cell lines.

## Conclusion

In this study, a cell-imprinted-based integrated microfluidic device was presented for biomedical applications, which improved the traditional imprinting cell-imprinted substrate efficiency by controlling the cell culture space. In this method, template cells were prepared in a regular pattern employing a microfluidic chip. After mold casting by PDMS, it was used as a cell-imprinted substrate under another microfluidic chip with the same pattern. When secondary cells were injected into the cell-imprinted-based integrated microfluidic device, there were only template cell patterns under them where they could be placed on it. In addition to PDMS, any materials with nanometer and micrometer dimensions can execute the process of making a cell-imprinted-based integrated microfluidic device based on the cell membrane topography.

In our method, cell culture was objective, non-random, and dynamic in contrast with traditional imprinting methods. Also, the cell culture medium continuously passed through cells.

Also, by applying microfluidic devices and reducing the experimental device’s size to micrometer levels, the amount of cell culture medium and the number of cells required in an experiment was reduced leads to a more economical process.

In the stem cell differentiation application, it was possible to supply more differentiated stem cells using a network of multiple integrated microfluidic devices connected to a syringe pump, which can be useful for future clinical trials.

As the topography of the cell-imprinted substrate is consistent with the target cells’ natural phenotype, the cells’ function on the cell-imprinted substrates is closer to their normal behavior in the body than on rigid polystyrene cell culture plates where substrate’s rigidity can increase the osteogenic differentiation probability^81^. So, this cheap and reproducible procedure can be used in all cell culture applications, such as growth and proliferation (while preserving normal cell activity) for drug analysis applications or differentiation for various tissue engineering and cell therapeutic applications.

Numerical simulation results can also be used as a guide to determine the effective factors in experimental conditions before entering the laboratory. The simulation results in this study showed that parameters such as injection speed, number, and size of cells, as well as channel dimensions, are effective in the experimental results, and suitable conditions were obtained using simulation before experimental analysis. Validation of simulation results with experimental results showed the power of numerical methods, which saves time and money. In fact, without attending the laboratory, we designed a virtual lab that does not require cell culture materials, the preparation of cell lines, the isolation of cells from the animals, and the operator for culturing, passage, and daily care of cells.

## Supplementary Information

Supplementary Information.

Supplementary Video 1.

Supplementary Video 2.
