# Supplementary material for: Computational and experimental studies of a cell-imprinted-based integrated microfluidic device for biomedical applications
Source: Sci Rep. 2021 Jun 9;11:12130. doi: 10.1038/s41598-021-91616-2 (PMC8190060; doi:10.1038/s41598-021-91616-2)
Supplement: Supplementary file 1 — Supplementary Information. [file 41598_2021_91616_MOESM1_ESM.docx]

Computational and experimental studies of a cell-imprinted-based integrated microfluidic device for biomedical applications

Sepideh Yazdian Kashani^a^, Mostafa Keshavarz Moraveji^a,^*, Shahin Bonakdar^b,^*

^a^ Department of Chemical Engineering, Amirkabir University of Technology (Tehran Polytechnic), 1591634311, Tehran, Iran

^b^ National Cell Bank Department, Pasteur Institute of Iran, P.O. Box 13169-43551, Tehran, Iran

*Corresponding authors:

Mostafa Keshavarz Moraveji: Department of Chemical Engineering, Amirkabir University of Technology (Tehran Polytechnic), 1591634311, Tehran, Iran; Tel.: +98 21 64543182; E-mail address: moraveji@aut.ac.ir

Shahin Bonakdar: National Cell Bank Department, Pasteur Institute of Iran, Tehran, Iran; Tel.: +98 21 64112359; Fax: +98 21 66492595; E-mail address: sh_bonakdar@pasteur.ac.ir


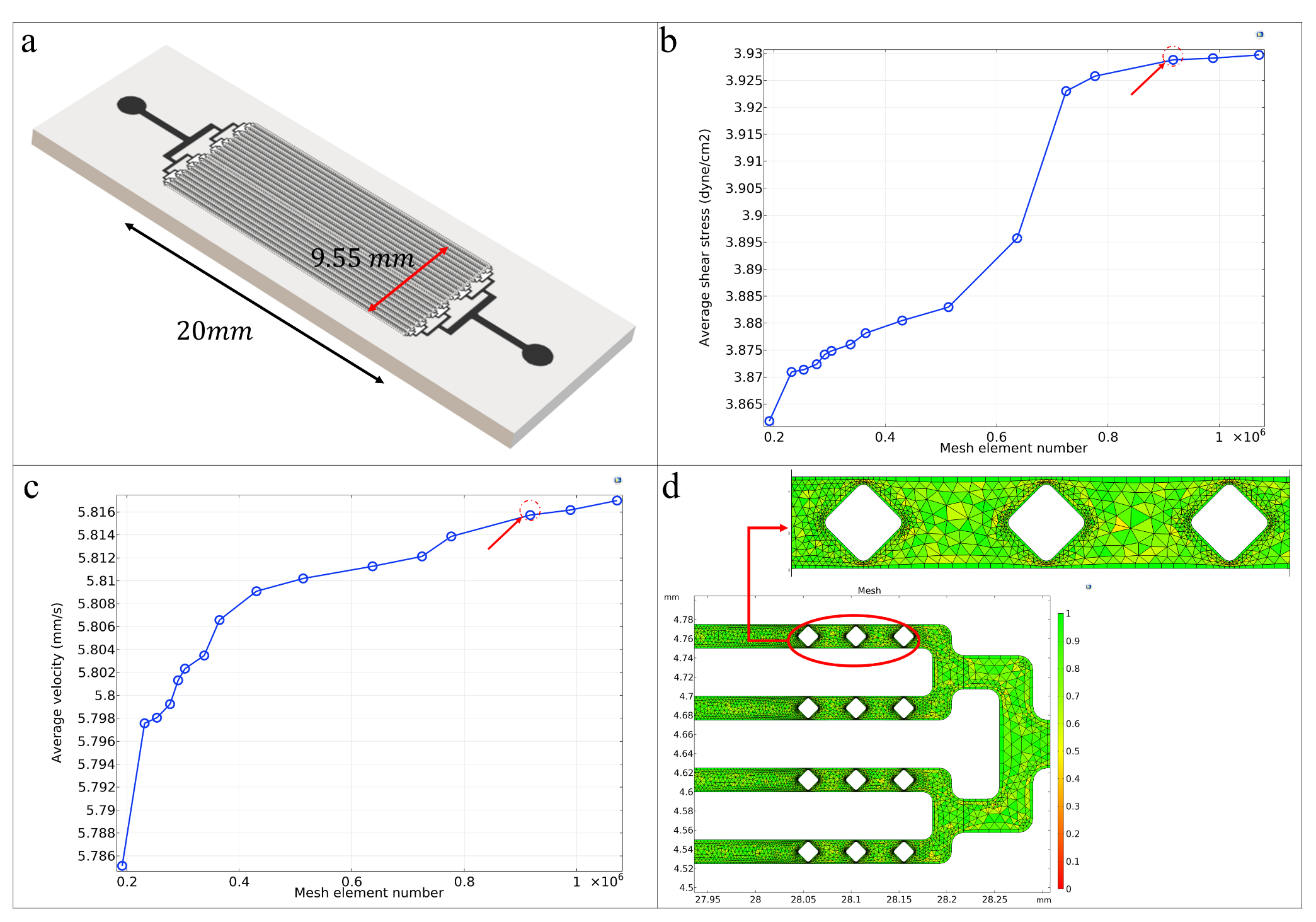


**FigS1.** a) Design of the microfluidic chip with 128 25 μm microchannels. b) Convergence study for average shear stress in the microfluidic chip. c) Convergence study for average velocity microfluidic chip. d) Mesh plot around terminal microposts at the end of each microchannel. The color bar shows the mesh quality.


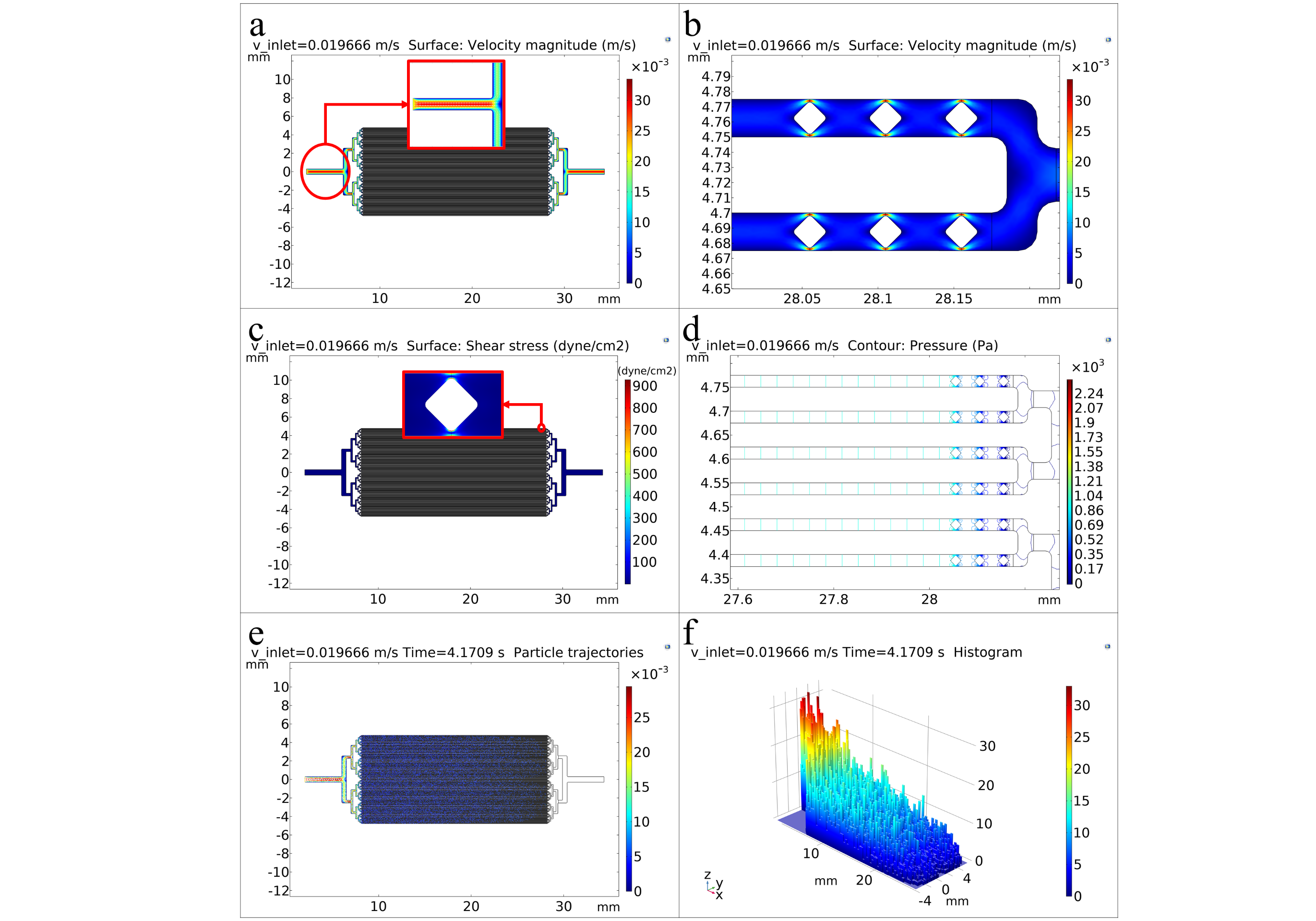


**FigS2.** a) Velocity profile in the microfluidic chip. b) Velocity profile in the microfluidic chip around terminal microposts. c) Shear stress profile in the microfluidic chip. The shear stress profile around a terminal microposts is zoomed. d) Pressure contour in the microfluidic chip around terminal microposts. e) Cell trajectories in the microfluidic chip when the first cells reach the terminal microposts. The cells' color indicates their velocity (m/s). A scale factor of 4 is used for cell radius for better demonstration. f) Histogram plot of cells distribution along the microfluidic chip when the first cells reach the terminal microposts. All data were obtained for the microfluidic chip with 25 μm microchannels


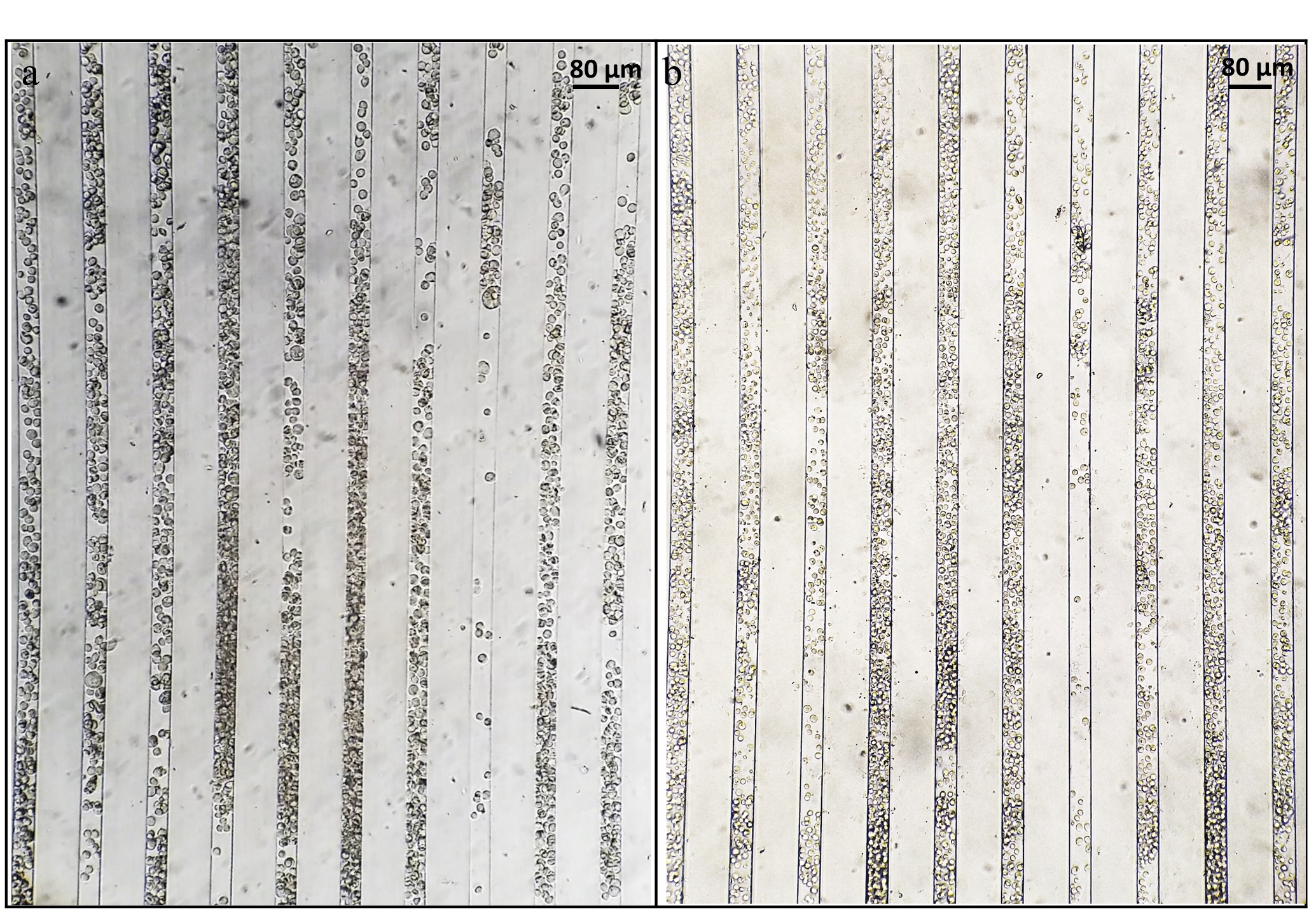


**FigS3.** a) 40µm microchannels of the microfluidic chip are almost filled with HUVEC cell line after injection with a syringe pump. b) 40µm microchannels of the microfluidic chip almost filled with chondrocytes after injection with a syringe pump.

**Supplementary video 1.** Cell trajectories animation in the microfluidic chip with 40µm microchannels (a scale factor of 4.9 is used for cell radius for better demonstration)

**Supplementary video 2.** Comparison of cell trajectories CFD simulation and experimental results
